# Sex Differences in 20-Hydroxyecdysone Hormone Levels Control Sexual Dimorphism in *Bicyclus anynana* Wing Patterns

**DOI:** 10.1093/molbev/msx301

**Published:** 2017-11-20

**Authors:** Shivam Bhardwaj, Kathleen L Prudic, Ashley Bear, Mainak Dasgupta, Bethany R Wasik, Xiaoling Tong, Wei Fun Cheong, Markus R Wenk, Antónia Monteiro

**Affiliations:** 1 Department of Biological Sciences, National University of Singapore, Singapore; 2 Department of Ecology and Evolutionary Biology, Yale University, New Haven, CT; 3 Cornell University Press, Cornell University, Ithaca, NY; 4 State Key Laboratory of Silkworm Genome Biology, Southwest University, Beibei District, Chongqing, China; 5 Department of Biochemistry, National University of Singapore, Singapore; 6 Yale-NUS College, Singapore

**Keywords:** sex hormone, insect, 20E, endocrinology, sexual traits, secondary sexual traits, sexual dimorphism, butterfly, *Bicyclus anynana*

## Abstract

In contrast to the important role of hormones in the development of sexual traits in vertebrates (Cox RM, Stenquist DS, Calsbeek R. 2009. Testosterone, growth and the evolution of sexual size dimorphism. *J Evol Biol.* 22(8):1586–1598.), the differentiation of these traits in insects is attributed almost exclusively to cell-autonomous mechanisms controlled by members of the sex determination pathway (Verhulst EC, van de Zande L. 2015. Double nexus – doublesex is the connecting element in sex determination. *Brief Funct Genomics* 14(6):396–406.), such as *doublesex*. Although hormones can shape the development of sexual traits in insects, variation in hormone levels are not conclusively known to cause dimorphism in these traits (Prakash A, Monteiro A. 2016. Molecular mechanisms of secondary sexual trait development in insects. *Curr Opin Insect Sci.* 17:40–48.). Here, we show that butterflies use sex-specific differences in 20-hydroxyecdysone hormone titers to create sexually dimorphic wing ornaments. Females of the dry season (DS) form of *Bicyclus anynana* display a larger sexual ornament on their wings than males, whereas in the wet season form both sexes have similarly sized ornaments (Prudic KL, Jeon C, Cao H, Monteiro A. 2011. Developmental plasticity in sexual roles of butterfly species drives mutual sexual ornamentation. *Science* 331(6013):73–75.). High levels of circulating 20-hydroxyecdysone during larval development in DS females and wet season forms cause proliferation of the cells fated to give rise to this wing ornament, and results in sexual dimorphism in the DS forms. This study advances our understanding of how the environment regulates sex-specific patterns of plasticity of sexual ornaments and conclusively shows that hormones can play a role in the development of secondary sexual traits in insects, just like they do in vertebrates.

## Introduction

Recent studies have shown that sexual traits are neither under constant, or even similar direction of selection over time and space (Cornwallis and Uller 2010; Stillwell et al. 2010; Miller and Svensson 2014). This is because organisms do not live in stable biotic and abiotic environments. One consequence of predictable and recurrent environmental changes, such as seasons, is the evolution of plasticity in sexual traits (Kajiura and Tricas 1996; Whitman and Ananthakrishnan 2009). Understanding the mechanisms behind the development of such plastic traits can help in developing better models of phenotypic evolution by focusing research on the actual genetic loci of evolution (Hoekstra and Coyne 2007).

**Fig. 1.**
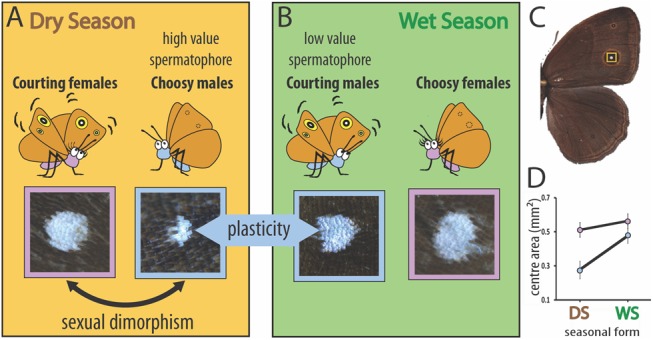
Sexual dimorphism and phenotypic plasticity in the size of dorsal eyespot centers in *Bicyclus anynana*. (*A*) Summary of the behavioral ecology and sexual ornament size of DS individuals and (*B*) WS individuals. (*C*) The eyespot centers (highlighted in yellow) are (*D*) sexually dimorphic in size in DS individuals (*F*_1, 37_ = 18.215, *P* < 0.001) and plastic in males across seasons (*F*_1, 37_ = 60.712, *P* < 0.001) (blue symbols/outlines = males; pink = females). Sizes along the *Y* axis apply to wings with an area of 208.805 mm^2^. *N* = 20 for each data point. Error bars represent 95% CI of means.

*Bicyclus anynana* butterflies evolved in a seasonal environment in Africa, experiencing predictable and recurrent dry and wet seasons (DS and WS) (Brakefield et al. 1996). As a consequence of this heterogeneity this species evolved a complex pattern of plasticity in its sexual behavior, sexual dimorphism in ommatidia size and opsin expression, as well as in the size of its sexual ornaments, the bright, UV-reflective dorsal eyespot centers (fig. 1) (Prudic et al. 2011; Everett et al. 2012; Macias-Muñoz et al. 2016). Essentially, DS individuals display sexual dimorphism in the size of the ornaments, with the courting DS females avidly displaying their unusually large sexual ornaments to the choosy cryptic males which have overall smaller eyespots (fig. 1) (Prudic et al. 2011). In the WS, both sexes develop large eyespots characteristic of the season and males avidly court choosy females. This leads to a pattern of sexual dimorphism in the DS and plasticity in the sexual ornament that is male-limited (fig. 1) (Prudic et al. 2011).

While the ultimate selective factors behind the patterns of sexual dimorphism and plasticity in ornament size in *B. anynana* are becoming increasingly clear (Prudic et al. 2011), the proximate factors behind these patterns are not understood. Here, we set out to examine the developmental mechanisms that regulate sexual ornament size dimorphism in DS individuals and male-limited plasticity in this butterfly species.

## Results

**Fig. 2.**
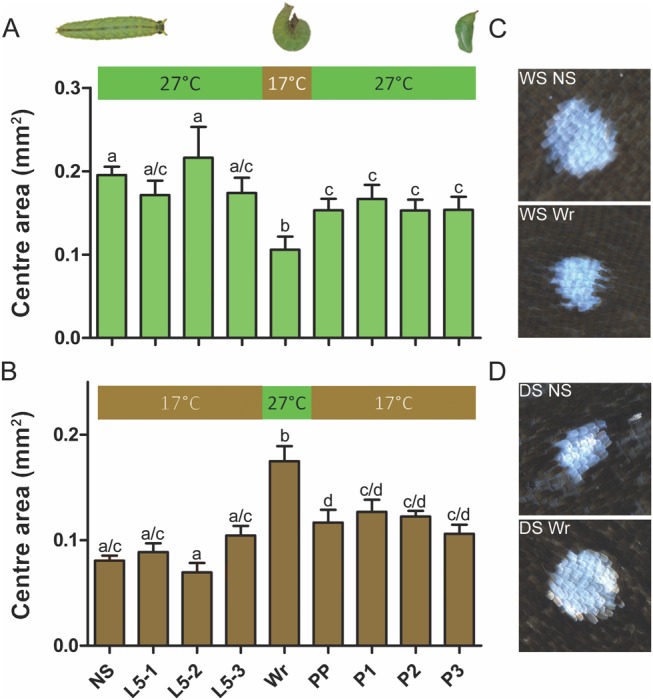
Temperature-shift experiments point to wandering (Wr) stage as the most important temperature-sensitive developmental stage for eyespot center size determination. Horizontal axis labels refer to the stage of development at the start of the 48-h shift; NS, nonshifted controls. L5 1–3 represent stages in larval 5th instar; Wr, wandering stage; PP, prepupal stage; P1–3 represent stages in pupal development. (*A*) Animals were reared at 27 °C throughout development, except for a 48-h window, where they were moved to a lower temperature of 17 °C. (*B*) Animals were reared at 17 °C throughout development, except for a 48-h window, where they were moved to a higher temperature of 27 °C. *N* = 20 for each data point. Error bars represent 95% CI of means. (*C*) Representative eyespot center images for nonshifted WS animals, contrasted with those shifted to 17 °C during Wr stage. (*D*) Representative eyespot center images for nonshifted DS animals, contrasted with those shifted to 27 °C during Wr stage. Groups that do not share the same letter superscripts are significantly different from each other.

Because ornament size in males is controlled by rearing temperature (Prudic et al. 2011), we began by identifying the developmental window that is critical for eyespot size regulation using temperature shift experiments. Low rearing temperature typical of the DS (17 °C) leads to DS butterflies, whereas high temperature typical of the WS (27 °C) leads to WS butterflies (Brakefield and Reitsma 1991). We experimentally manipulated rearing temperature for brief windows of 48 h at different stages of development by moving animals from one temperature to the alternate temperature, and then returning them back to the original temperature (fig. 2). WS animals reared at 27 °C, which were moved to 17 °C during the wandering (Wr) stage of larval development showed the strongest decrease in eyespot size (fig. 2*A* and *C*). The opposite pattern, an increase in eyespot size, was seen in animals reared throughout at 17 °C, and moved briefly to 27 °C for a 48-h interval during the same Wr stage (fig. 2*B* and *D*). These experiments show that the Wr stage is critical for the determination of dorsal eyespot center size in males. Therefore, we focused our subsequent investigations of eyespot center size around this developmental stage.

**Fig. 3.**
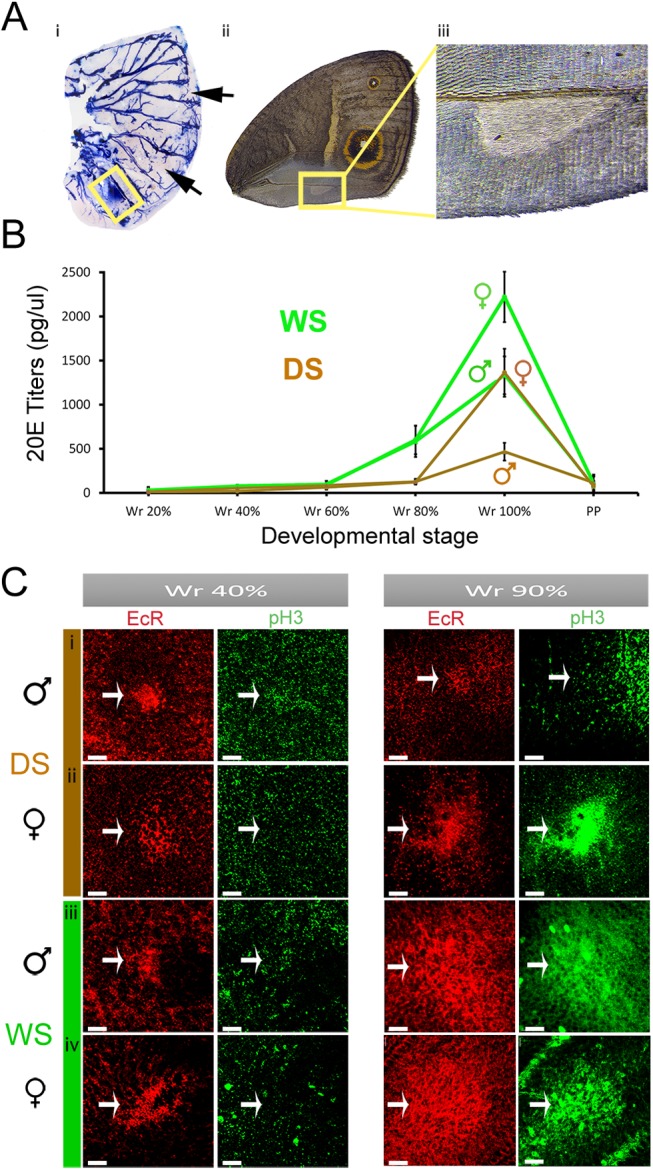
Sexually dimorphic 20-hydroxyecdysone titers, but not *doublesex* isoforms, are associated with cell division and larger EcR expression domains in late Wr stage eyespot centers. (*A*) (i) *dsx* mRNA is present in the pheromone producing organ of males (yellow box) but is absent from the eyespot centers (arrows). *N* = 4 for in situ stainings*.* (ii) Male forewing with male pheromone producing organ (iii). (*B*) 20E titers observed during fine intervals of wandering (Wr) and prepupal (PP) stages. Error bars represent 95% CI of means. (*C*) Larval wings immunostained with EcR (Red) and pH3 (Green) antibodies at two stages of Wr stage—40% and 90% development, zoomed in to show the developing dorsal Cu1 eyespot centers (fig. 1*C*). Scale bars, 20 μm.

Previous studies on the developmental basis of sexual traits in insects have pointed exclusively to cell-autonomous mechanisms involving the activation of members of the sex-determination pathway, such as the gene *doublesex* (*dsx*), in the cells that develop the trait (Tanaka et al. 2011; Prakash and Monteiro 2016). Therefore, we asked whether *dsx* was being expressed in the eyespot centers at the wandering stage of development. In situ hybridizations with a probe generated against a common region of *dsx* (i.e., made to identify both male and female isoforms of this gene) identified *dsx* expression in the developing androconial organs, a sex-pheromone producing organ (Costanzo and Monteiro 2007; Nieberding et al. 2008; Dion et al. 2016) in the wings of males (fig. 3*A*). However, no *dsx* expression could be detected in the developing eyespot centers of Wr larvae (fig. 3*A* and supplementary fig. 3, Supplementary Material online).

This led us to ask whether the sexual ornaments could be under the control of sex-specific hormone titers. Previous studies have implicated insect hormones in the development and maintenance of sexual traits in insects (Prakash and Monteiro 2016), but to date no study to our knowledge has ever shown sexual dimorphism in hormone titers leading to the development of sexual traits in insects. Furthermore, previous research in this species showed that levels of the molting hormone, 20-hydoxyecdysone (20E), were involved in regulating ventral eyespot center size in females during the Wr stages of development. We, therefore, asked whether levels of this hormone could be sexually dimorphic at the Wr stage.

We collected hemolymph from developing male and female larvae at finely spaced intervals during the Wr stage, and observed a rise in 20E titers in all WS and DS forms toward the end of this stage, just before the Wr larvae turned into prepupae. Furthermore, male and female 20E titers were sexually dimorphic within each seasonal form, with females having higher titers than males (*F*_1, 41_=55.78, *P* < 0.001) (fig. 3*B*). In addition, WS titers were higher than DS titers, as previously reported for females (Oostra et al. 2014; Monteiro et al. 2015) (*F*_1, 41_=52.11, *P* < 0.001), with no interaction between season and sex (*F*_1, 41_=0.001, *P* = 0.977).

Steroid hormones such as 20E exert effects on cells only if such cells express correspondent hormone receptors (Stanisic et al. 2010). We looked for the presence of the Ecdysone Receptor (EcR) at two different stages during the Wr stage, an early stage (∼40% development) and a later stage (∼90% development), flanking the period before and after the rise in 20E tiers. At the early Wr stage, EcR was expressed in the dorsal eyespot centers in a similar extent in each sex and seasonal form (fig. 3*C* i–iv: panel 1), confirming the ability of these cells to respond to the subsequent rising titers of 20E, and the potential for this hormone to impact the developmental fate of these cells. At the later Wr stage, however, we observed a difference in the extent of EcR staining. DS males still expressed EcR in a small group of cells, whereas DS females and both WS sexes expressed EcR in a larger cluster of cells (fig. 3*C* i–iv: panel 3). This suggests that the size control of the sexual ornament appears to be taking place in between these two time points, primarily via an increase in cell number.

20E levels above certain thresholds are known to promote cell division in larval wing imaginal discs (Koyama et al. 2004; Herboso et al. 2015). Therefore, to visualize whether such localized cell divisions were taking place in the region of the future sexual ornaments, we studied the localization of a mitotic marker, phospho-histone H3 (pH3) (Juan et al. 1998), using fluorescently labeled anti-pH3 antibodies in the wing discs. At 40% of the Wr stage, when the 20E titers are low, we observed no pH3 staining (green, fig. 3*C* i–iv: panel 2). However, at the later stage (90% Wr), when 20E titers are surging, cell division was taking place in all groups, except DS males (fig. 3*C* i–iv: panel 4). We hypothesized that cell division is initiated only once a critical threshold of 20E is attained. The cells making up the sexual ornament of DS males, having the lowest 20E titers, may never reach this threshold, and hence do not experience 20E signaling at similar levels as the other groups, and do not divide.

**Fig. 4.**
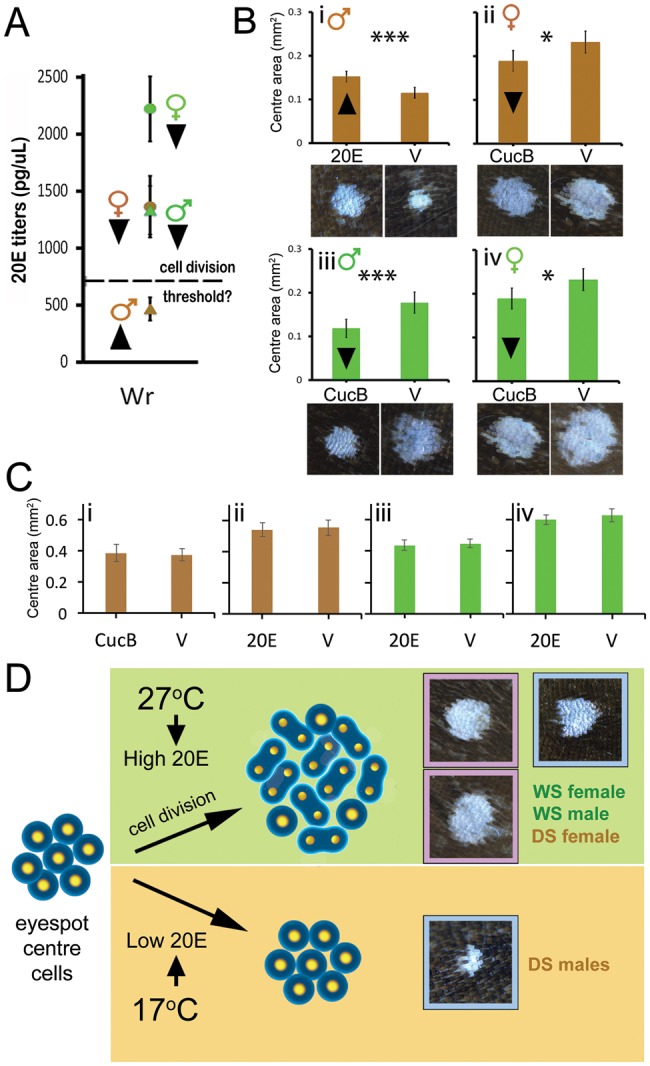
20E signaling promotes an increase in eyespot center size. (*A*) 20E titers in developing larvae at end of Wr stage. Dashed line represents hypothetical threshold of 20E titers required for cell division. Arrowheads next to data points represent planned manipulations to 20E signaling. (*B*) 20E injections cause an increase in eyespot size in DS males (i), whereas reduced EcR signaling using CucB causes a decrease in eyespot size in all other groups (ii–iv). Figures below respective graphs represent representative images obtained after treatments. (*C*) Opposite-direction hormone treatments (to the arrowheads in (*A*)) does not produce any significant differences in DS males (i), DS females (ii), WS males (iii), and WS females (iv), supporting the threshold-response hypothesis for cell division. Error bars represent 95% CI of means. (*D*) Diagram summarizing the interpretation of our results: Rearing temperature induces variation in 20E titers at the Wr stage of development. High titers result in cell division and larger eyespot centers, whereas low titers result in smaller centers, as seen in DS males (blue outlines = males; pink = females). DS females, despite being reared at low temperature, have sufficiently high 20E levels to also undergo cell division of the wing ornament.

To test this hypothesis, we manipulated 20E signaling in the four butterfly groups. We elevated 20E signaling in DS males by injecting them with 20E at ∼60% of the Wr stage; and lowered 20E signaling in the other three groups by injecting individuals with a EcR antagonist, Cucurbitacin B (CucB) (fig. 4*A*) (Dinan et al. 1997). Injections of 20E caused an increase in eyespot center size in DS males, relative to injections with vehicle (fig. 4*B* i; DS M- *F*_1, 37_=18.38, *P* < 0.01), whereas injections of CucB significantly reduced the eyespot center size in the other three groups relative to injections with vehicle (fig. 4*B* ii–iv; DS Fem: *F*_1, 46_=6.43, *P* = 0.015, WS Mal: *F*_1, 44_=13.75, *P* = 0.001, WS Fem: *F*_1, 37_=4.617, *P* = 0.038), indicating a functional role of 20E signaling in dictating the size of these sexual ornaments.

To further test the 20E threshold hypothesis, we manipulated 20E signaling in opposite directions, that is, increased 20E titers for groups already having high 20E titers, and reduced 20E signaling in DS males, which were supposedly already below the threshold level of 20E that leads to eyespot center cell division. We did not observe any significant eyespot center size increase or decrease across treatments in all four groups (fig. 4*C* i–iv), indicating that 20E levels above a threshold value are indeed necessary and sufficient for the induction of cell division and eyespot center size determination.

## Discussion

In summary, here we have shown that sex-specific levels of a steroid hormone, during a brief period of development, controls a very localized pattern of division in cells that express the hormone receptor, which later develop into the bright UV-reflective scale cells that make up a sexual ornament in adult butterflies. Females produce more of this hormone than males, and WS forms more than DS forms. However, all groups, except DS males, produce sufficient hormone to trigger a process of local cell division at the center of the dorsal eyespots. This creates sexual dimorphism in ornament size in DS animals, and plasticity in ornament size in males.

Sexual dimorphism in some vertebrate traits, such as the length of digits in mice, is controlled by two hormones, androgen and estrogen steroids, present in different relative amounts in each sex during a small window of development (Zheng and Cohn 2011). Our study indicates that sexual dimorphism can be achieved via the use of a single hormone, 20-hydroxyecdysone, present in each sex at different levels.

It is likely that this butterfly species, which has evolved a complex mechanisms for the regulation of plasticity in the size of its ventral eyespots (Brakefield et al. 1996; Monteiro et al. 2015), which function in predator–prey interactions (Lyytinen et al. 2004; Prudic et al. 2015), simply co-opted this mechanism to also regulate the size of its dorsal eyespots. The selection pressures working on dorsal eyespots, however, are different from those on ventral eyespots; so, the mechanism of plasticity had to be tweaked to allow eyespots on different surfaces to display different reaction norms for size in response to environmental temperature. Part of the tweaking appears to have been the rise in hormone titers in DS females relative to DS males, allowing females to develop large dorsal eyespots in the DS. Why DS females are able to maintain small ventral hindwing eyespots requires further experiments. Additional work will also be necessary for a better understanding of how and when the sexual dimorphism in the hormone titers actually evolved. It is likely that the sexual dimorphism observed in ecdysone receptor expression in the late wandering stage of eyespot development of DS forms merely reflects the process of cell division that takes place after the 20E titers surge in DS females, but not in DS males.

An important advance of this work is the demonstration that different levels of a steroid hormone in an insect control sexually dimorphic traits. Previous reports proposed that sexually dimorphic traits (such as horn length in *Onthophagus* and wing dimorphism in *Planococcus kraunhiae)* could be under hormonal regulation (Emlen and Nijhout 1999; Emlen et al. 2005; Vea et al. 2016), or implicated hormones in the maintenance of sexual dimorphism of adult insects (Fagegaltier et al. 2014), but no study has conclusively reported different levels of insect hormones as key developmental regulators of sexual traits (Prakash and Monteiro 2016). Sexual trait determination in insects has, so far, been attributed exclusively to cell-autonomous mechanisms involving the expression of sex-specific splice variants and factors from the sex determination pathway, such as Feminizer (Fem), Transformer (Tra), Fruitless (Fru), and Doublesex (Dsx), in cells that build the sexually dimorphic trait (Keyes et al. 1992; Gempe et al. 2009; Prakash and Monteiro 2016). Here, we show conclusively, that sexual differences in hormone titers regulate dimorphic sexual traits, instead of cell-autonomous factors.

Sexual trait development in insects has, thus, been considered distinct from sexual trait development in vertebrates, where steroid hormones, such as testosterone and estrogen, are important regulators of sexual dimorphism (Schlinger 1997; Zheng and Cohn 2011). Until recently, hormones were considered the exclusive means by which vertebrates regulate their sexual traits (Bear and Monteiro 2013), but the appearance of gynandromorphic finches (Agate et al. 2003), displaying half male and half female plumage patterns, finally led researchers to consider the presence of cell-autonomous mechanisms of sexual trait development in vertebrates. The striking appearance of gynandromorphic insects (Morgan, Bridges, Sturtevant 1919), in turn, led most biologists to assume insects used cell-autonomous processes exclusively to differentiate sexual traits. Our work now conclusively shows that both mechanisms are playing a role in vertebrates and insects and calls for additional comparative work to understand how these two convergent mechanisms of sexual trait development may have diversified and evolved.

## Materials and Methods

### Butterfly Husbandry

*Bicyclus* *anynana* butterflies, originally from Malawi, were reared in two climate rooms at 17 °C and 27 °C, at 70% relative humidity, 12:12 h light:dark cycle, to produce the dry and wet season forms, respectively. Larvae were fed young corn, whereas adults were fed ripe mashed banana.

### Eyespot and Eyespot Center Size Measurements

*Bicyclus* *anynana* adults from each season and sex were dissected and imaged using a Leica Stereo Microscope. Area measurements for dorsal forewings, individual posterior Cu1 eyespot, and white centers were calculated using ImageJ (NIH, v1.45s), as described previously(Monteiro et al. 2015).

### Wandering Stage Sampling

Late fifth instar larvae were kept with ample food in transparent containers and imaged at 5-min intervals using the time-lapse feature of a RICOH Pentax WG-3 Camera, using method described previously (Monteiro et al. 2015). Initiation of wandering stage happened when the larvae left the food and started wandering up. End of wandering stage happened when the animal begun hanging from the container, upside down.

### *Doublesex* In Situ Hybridization

A fragment of *doublesex* mRNA from *B. anynana* was amplified from the cDNA using the primers AM0016 (5′-GGTGTCCGTGGGCCCGTG-3′-forward) and AM0017 (5′-CCGGTCCAGCTCCAGGCG-3′-reverse) and cloned into the pGEMT-Easy vector (Promega). See supplementary figure 1, Supplementary Material online, for the position of the probe and primers. The insert was amplified using universal M13 primers and the amplicon was used as a template to synthesize DIG-labeled RNA probes. Wing discs were collected from the Wr stage larvae and used for RNA in situ hybridization as described previously (Martin and Reed 2014). A Leica stereo microscope was used for imaging the stained tissues.

### Semiquantitative RT-PCR

To complement our findings from the in situ hybridization, we performed semiquantitative RT-PCR in two different sectors of the wings of Wr larvae. Late Wr stage larval wing discs were extracted and dissected into a proximal and a distal sector (supplementary fig. 2, Supplementary Material online). Proximal sectors contain the male androconial organ and hair pencils (only in hindwings), whereas distal sectors contain the sexually dimorphic eyespots. Wings were stored in TRIzol reagent (Life Technologies, Cat No. 15596-018) at −80 °C immediately after dissection. Extracted wing tissues were homogenized in TRIzol using a bullet blender, followed by a chloroform-isopropanol precipitation and ethanol wash. Subsequently, we treated extracted RNA with DNAse, and incubated at 37 °C for 15 min, followed by 3 M NaoAC treatment and incubation at −80 °C for precipitation. Extracted RNA was followed through one round of phenol–chloroform RNA extraction. We then used 500 ng of RNA from each tissue sample to do a reverse transcription by adding dNTPs, Reverse transcriptase, and RNAse inhibitor at 42 °C for 1 h to generate cDNA. A fragment of *doublesex* was amplified from this cDNA using the primers AM0462 (5′-AGTACCGCTTGTGGCCCTTC-3′-forward) and AM0463 (5′-GTCCGCGTGCGAAATACATC-3′-reverse). We used a housekeeping gene, EF-1α, as an internal control, which was amplified using primers AM0110 (5′-GTGGGCGTCAACAAAATGGA-3′-forward) and AM0111 (5′-GCAAAAACAACGAT-3′-reverse).

Male proximal forewing sectors, containing the androconial organ, expressed *doublesex*, whereas distal forewing sectors containing eyespots, completely lacked *doublesex* expression at this stage in development. Females, which lack the androconial organ, lacked *dsx* expression in both proximal and distal sectors. In addition, we observed similar expression patterns of *dsx* in hindwing anterior and posterior sectors. Anterior sectors, which contain androconial organs and hair pencils in males, show presence of *dsx*, which is absent in posterior sectors with eyespots. These results reinforce the idea that *doublesex* is not involved in regulating sexual dimorphism in eyespots.

### Hemolymph Collection

A small puncture was made to the first abdominal proleg of individual wanderers, and prepupae, and 20 μl of hemolymph were collected using a pipet. Hemolymph collections were taken from WS and DS male and female wanderers at five time points following the onset of wandering (20%, 40%, 60%, 80%, and 100%), and from prepupae (at 2 PM after the onset of prepupae). *N* = 4 per time point per seasonal form, but *N* ≥ 12 for Wr 80 and Wr 100%. Sample preparation followed an established protocol (Westerlund and Hoffmann 2004).

### Hormone Extraction

We added 800 μl of HPLC grade water to the 200 μl sample of 20 μl of hemolymph + 45 μl methanol + 45 μl iso-octane and then vortexed the solution. We used a previously described protocol (Monteiro et al. 2015).

### Hormone Titer Measurements using UPLC/MS

About 20 μl of sample was transferred into sample vial and 5 μl of 250 μg/ml deuterated-2, 2, 4, 4-chenodeoxycholic acid (Catalogue No. DLM-6780-PK, Cambridge Isotopes Laboratories, Andover, MA) (additional internal control against loss of MS sensitivity upon repeated exposure) was spiked into the sample (to make a final concentration of 50 μg/ml d4-chenodeoxycholic acid as internal standard). A series concentration of 20-hydroxyecdysone commercial hormone (Sigma–Aldrich, Catalogue No. H5142, Lot No. 060M1390V) (1, 2, 5, 8, and 10 µg/ml) were all spiked with a constant amount of d4-chenodeoxycholic acid (50 µg/ml) and analyzed via LC-MS on an Agilent 1100 LC system coupled with an ABSciex 4000 QTrap mass spectrometer. Liquid chromatography was performed on an Eclipse XDB-C18, 5 µm, 4.6 mm × 150 mm column (Agilent Technologies Corp, Santa Clara CA). HPLC conditions: injection volume 10 μl; mobile phase A and B consisted of reverse osmotic water and methanol, both containing 0.1% of formic acid; flow rate 0.5 ml/min, 30% B for 0.1 min, and linearly changed to 80% B in 0.2 min; then linearly switched to100% B in 1.2 min and maintained for 1.3 min, and then linearly changed to 30% B in 2.6 min and maintained for 7.4 min. Then, the flow rate and the mobile phase were returned to the original ratio. Mass spectrometry was recorded under the positive ESI mode. A blank injection of 100% MeOH was run after each sample injection to ensure no carry over. Response factor (*F*) of commercial hormone to the internal standard, d4-chenodeoxycholic acid was determined. The linear range of detection for each standard was determined via the LC-MRM parameters. The result of a standard titration at 1, 2, 5, 8, and 10 µg/ml were subjected to linear regression analysis, and the correlation coefficient (*R*^2^). Lipids of hormone samples were measured using the validated LC-MRM parameters. Approximate concentration of butterfly hormone was calculated using the peak area under the curve. Intensity of individual hormone species was quantified by normalizing against the respective calibration curve of standards and labeled steroid.

### Ecdysone Receptor and pH3 Immunostainings

Wing discs were dissected from wanderers at different stages. Monoclonal (mouse) antibodies raised against a *Manduca sexta* EcR peptide shared across all EcR isoforms (Developmental Studies Hybridoma Bank, No. 10F1) (Jindra et al. 1996) were used at a concentration of 1:5. Goat antimouse (Molecular Probes, No. A-11001) was used as secondary antibody at a concentration of 1:800. Polyclonal antibodies raised against rabbit mitosis marker antiphospho-histone H3 (Ser 10) was used at a concentration 1:150 (Merck Milipore, No. 06-570). Goat antirabbit (Molecular probes, No. A-11034) was used as a secondary antibody at the concentration of 1:800. Wings were dissected, fixed in PFA, dehydrated in MeOH at −20 °C, rehydrated using a gradient of MeOH and water, and then treated with primary and secondary antibodies. All wings were double immunostained with pH3 and EcR, and mounted with ProLong Gold (Invitrogen, Carlsbad, CA). Images were captured on a LSM 510 META confocal microscope (Carl Zeiss, Jena, Germany). Serial Z-optic sections were done in order to distinguish dorsal from ventral EcR expression. At least three biological replicates were obtained for each immunostaining.

### Hormone Injections

Male DS wanderers (60% Wr) were injected with 4 μl of 2000 pg/μl of 20E (8000 pg total) (Sigma–Aldrich, Catalogue No. H5142, Lot No. 060M1390V) or 4 μl of vehicle (1 ethanol:9 saline solution). Female DS and male WS wanderers (60% Wr) were injected with 3 μl of 5600 pg/μl of cucurbitacin B (16, 800 pg total) (Sigma–Aldrich, Catalogue No. C8499, Lot No. 035M47104V) or 3 μl of vehicle (1 ethanol:9 saline solution). Female WS wanderers (60% Wr) were injected with 4 μl of 5600 pg/μl of cucurbitacin B (22, 400 pg total) or 4 μl of vehicle (1 ethanol:9 saline solution). To test the threshold hypothesis, DS male wanderers were injected with 3 μl of 5600 pg/μl of cucurbitacin B (16, 800 pg total) and DS females, WS males and females were injected with 4 μl of 2000 pg/μl of 20E (8000 pg total). Control injections were made for all groups with vehicle (1 ethanol:9 saline solution). All solutions were stored at −20 °C. The injections were done using a Hamilton syringe (10 μl 700 series hand fitted microliter syringe with a 33 gauge, 0.5-inch needle). The injection site was on the dorsal surface in between the integument of the second and third thoracic leg after the larvae had been chilled for 30 min on ice.

### Statistical Analyses

Eyespot center size was compared across seasonal forms or treatments using analyses of covariance (ANCOVA), where wing area was used as a covariate. Fixed factors appearing in the model were evaluated at a wing area of 175.265 mm^2^ for WS and 193.021 mm^2^ for DS wings. Hemolymph titers were compared using two-way ANOVAs with seasonal form and sex as fixed factors. All analyses used the GLM procedure in SPSS Statistics (version 19). Data were log-transformed to meet homogeneity of variance criteria (as determined by a Levene’s test). Pair-wise comparisons, using a Bonferroni correction for multiple comparisons, were used to detect which developmental time switch points produced significant differences in eyespot traits in the temperature-shift analyses. Graphs were made in Microsoft Excel (version 14.6.5 for the Mac) and Adobe Illustrator CC2015 using reverse transformed data (when applicable).

## Supplementary Material

Supplementary data are available at *Molecular Biology and Evolution* online.

## Author Contributions

Conceived and designed the experiments: S.B., K.L.P., and A.M. Performed the experiments: S.B., K.L.P., A.B., M.D.G., B.R.W., X.T., and W.F.C. Analyzed the data: S.B., W.F.C., M.R.W., and A.M. Wrote the paper S.B. and A.M.

## Supplementary Material

Supplementary Data
