# Correction: Sex Differences in 20-Hydroxyecdysone Hormone Levels Control Sexual Dimorphism in *Bicyclus anynana* Wing Patterns

**DOI:** 10.1093/molbev/msad127

**Published:** 2023-06-06

This is a correction to: Shivam Bhardwaj and others, Sex Differences in 20-Hydroxyecdysone Hormone Levels Control Sexual Dimorphism in *Bicyclus anynana* Wing Patterns, *Molecular Biology and Evolution*, Volume 35, Issue 2, February 2018, Pages 465–472, https://doi.org/10.1093/molbev/msx301

In the originally published version of this manuscript, there was a potential image irregularity in Panel B in Figure 4. The Figure should read:

**Figure:**
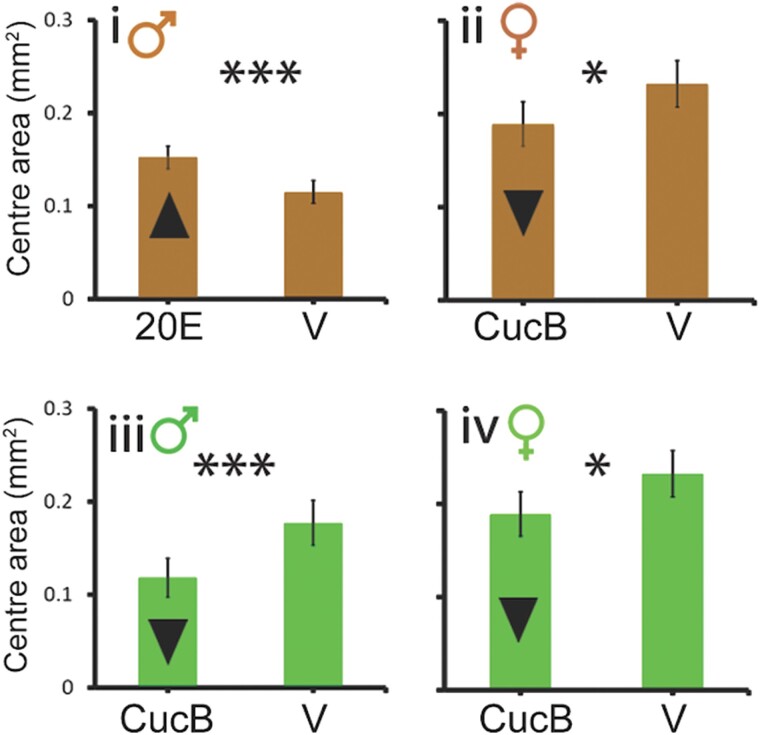


instead of:

**Figure:**
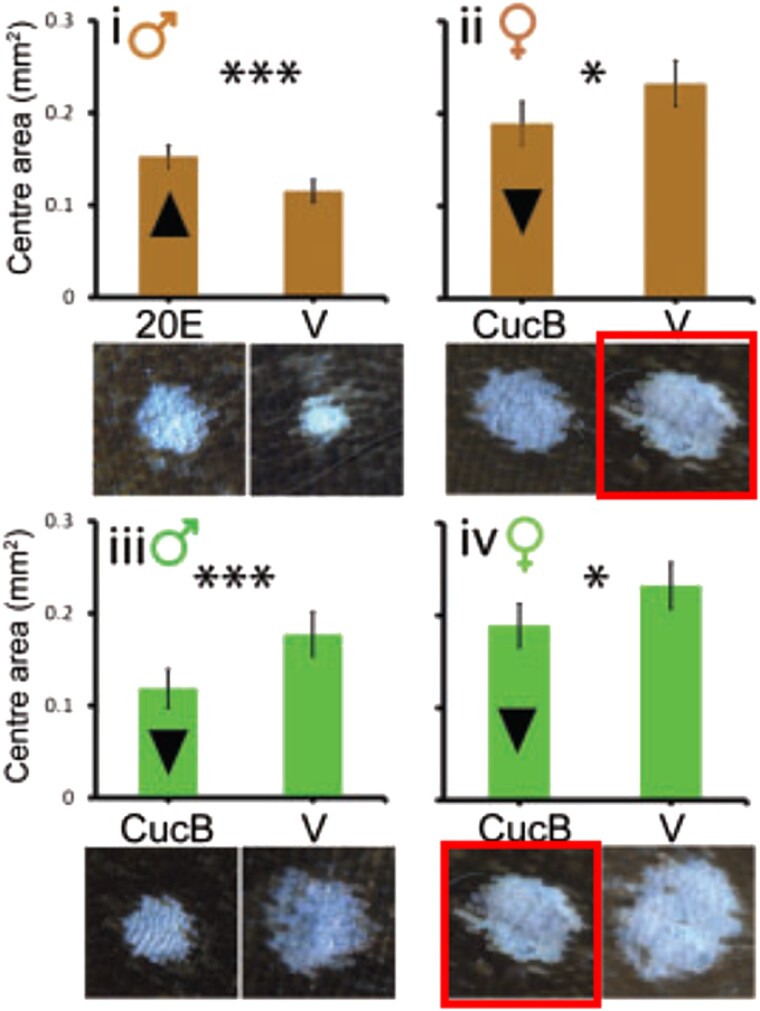


A Table is added here also (Supplementary Table S1) indicating raw data that were used to plot Fig 3B and Fig 4A (late Wr stage).

These emendations are outlined only in this correction notice to preserve the version of record.

