# Supplementary material for: Sex Differences in 20-Hydroxyecdysone Hormone Levels Control Sexual Dimorphism in Bicyclus anynana Wing Patterns
Source: Mol Biol Evol. 2017 Nov 20;35(2):465–72. doi: 10.1093/molbev/msx301 (PMC5850599; doi:10.1093/molbev/msx301)
Supplement: Supplementary Data [file msx301_suppl.pdf]

Supplementary information

A

```
B.mori-Dsx-M     CCCAGCAATGGTGTCGATGGGCTCATGGAAACGCCGCGTGCCCGATGACTGCGAGGAGCG
B.mori-Dsx-F     CCCAGCAATGGTGTCGATGGGCTCATGGAAACGCCGCGTGCCCGATGACTGCGAGGAGCG
B.any-Dsx        -------ATGGTGTCCGTGGGCCCGTGGCGGCGCCGCACGCCCGACGAATGCGACGAGCG
                        ********  ***** * ***   ******  ****** ** ***** *****

B.mori-Dsx-M     TTCCGAACCCGGCGCCTCCAGTTCGGGCGTTCCTCGTGCCCCTCCTAACTGTGCGCGCTG
B.mori-Dsx-F     TTCCGAACCCGGCGCCTCCAGTTCGGGCGTTCCTCGTGCCCCTCCTAACTGTGCGCGCTG
B.any-Dsx        CTCCGACCCCGGCGCCTCCAGCTCGGGCGTGCCGCGCGCCGCCCAACTGTGCGCGCTG
                  ***** ************** ******** ** ** ** ** ** **************

B.mori-Dsx-M     CCGCAATCACAGGCTCAAGATCGAGCTCAAGGGTCACAAGCGGTACTGCAAATATCAGCA
B.mori-Dsx-F     CCGCAATCACAGGCTCAAGATCGAGCTCAAGGGTCACAAGCGGTACTGCAAATATCAGCA
B.any-Dsx        CCGCAACCACCGGCTCAAGATCGAGCTGAAGGGCCACAAGCGGTACTGCAAGTACCGGTA
                 ****** *** **************** ***** ***************** ** * * *

B.mori-Dsx-M     TTGCACTTGTGAAAAGTGCCGCCTTACCGCAGACAGGCAGCGGGTAATGGCAAAGCAAAC
B.mori-Dsx-F     TTGCACTTGTGAAAAGTGCCGCCTTACCGCAGACAGGCAGCGGGTAATGGCAAAGCAAAC
B.any-Dsx        CTGCACGTGCGAGAAGTGCCGGCTGACGGCCGACCGGCAGCGGGTCATGGCGCTGCAGAC
                  ***** ** ** ******** ** ** ** *** ********** *****   *** **

B.mori-Dsx-M     GGCAATCAGACGGGCCCAGGCTCAGGACGAAGCGCGTGCGCGTGCGCTGGAATTAGGAAT
B.mori-Dsx-F     GGCAATCAGACGGGCCCAGGCTCAGGACGAAGCGCGTGCGCGTGCGCTGGAATTAGGAAT
B.any-Dsx        GGCGCTGCGGCGTGCCCAGGCGCAGGACGAGGCGCGCGCGCGGGCGCGGGGCGGCGGGCC
                 ***  *  * ** ******** ******** ***** ***** **** **     **

B.mori-Dsx-M     ACAGCCCCCGGGGATGGAGTTGGATAGGCCTGTACCACCAGTGGTGAAGGCGCCACGCAG
B.mori-Dsx-F     ACAGCCCCCGGGGATGGAGTTGGATAGGCCTGTACCACCAGTGGTGAAGGCGCCACGCAG
B.any-Dsx        CGA---CCCGCGCCTGGAGCTGGACCGGCCCGAGCCGCCCGTGGTGAAGGCGCCGCGCAG
                   *   **** *  ***** ****  **** *  ** ** ************** *****
```

B

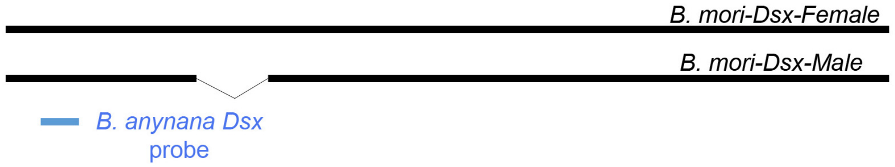

Supplemental Fig S1. Partial *dsx* sequence alignment in *B. anynana* and *in situ* probe location. (A) Partial alignment of the male and the female isoforms of *B. anynana dsx* coding sequence. The forward and the reverse primers used to amplify a region of the *dsx* sequence for RNA *in-situ* hybridization are highlighted in yellow. Note that the amplified fragment is common to both the female and the male isoforms. (B) Schematics showing the position of *B. anynana dsx* probe relative to the male and female isoforms of *dsx* from *B. mori*.

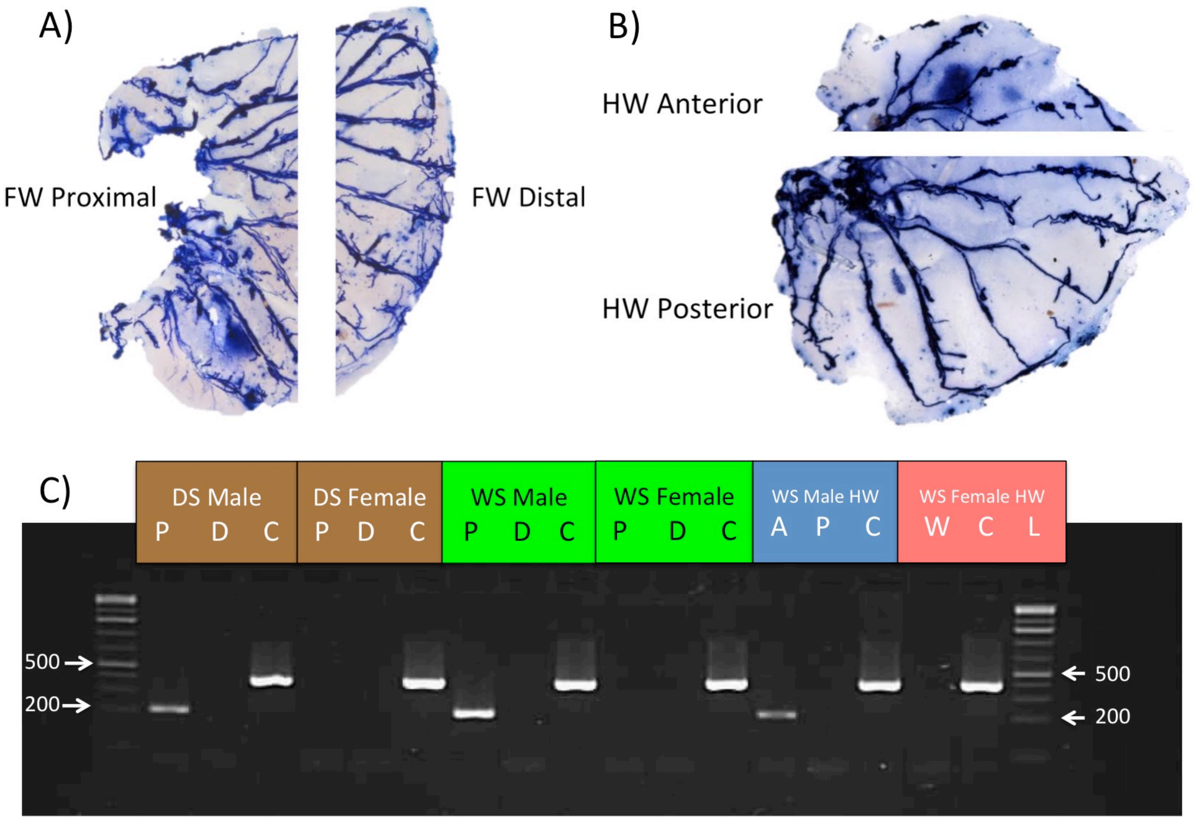

Supplemental Fig S2 (To fig. S3A). *Dsx* is not expressed in the developing eyespot centers in *B. anynana*, but is present in male androconial organs. (A) Proximal and distal forewing (FW) sectors in B. anynana (B) Anterior and posterior hindwing (HW) sectors. FW proximal and HW anterior sectors in males have androconial organs, which are absent in females. (C) Proximal sectors in FW in DS males and WS males express dsx. Similar expression in observed in WS male HW anterior sectors, which also contain androconial organs. dsx is absent in wing regions with eyespots in both males and females. EF-1α is present as a control in all treatments. (P- Proximal, D- Distal, C- Control, A-Anterior, P-Posterior, W-entire wing, L-1Kb plus ladder; dsx expected band size ~ 200bp, EF1α~ 450bp). We used three biological pools (Number of wings in each pool = 5) of males and females of each seasonal form for these experiments and the results were identical across all biological replicates.
